# Contraceptive method mix and preference: A focus on long acting reversible contraception in Urban Cameroon

**DOI:** 10.1371/journal.pone.0202967

**Published:** 2018-08-23

**Authors:** Atem Bethel Ajong, Philip Nana Njotang, Bruno Kenfack, Marie José Essi, Martin Ndinakie Yakum, Francklin Brice Soung Iballa, Enow Robinson Mbu

**Affiliations:** 1 Department of Obstetrics and Gynaecology, Faculty of Medicine and Biomedical Sciences, University of Yaoundé I, Yaoundé, Cameroon; 2 Obstetrics and Gynaecology unit, Yaoundé Central Hospital, Yaoundé, Cameroon; 3 Department of Biomedical Sciences, Faculty of Science, University of Dschang, Dschang, Cameroon; 4 Department of Public Health, Faculty of Medicine and Biomedical Sciences, University of Yaoundé I, Yaoundé, Cameroon; 5 Meilleur Accès aux Soins de Santé, Yaoundé, Cameroon; 6 Malaria Consortium-Cameroon Coalition Against Malaria (MC-CCAM), Global Fund, Bamenda, Cameroon; 7 Directorate of Family Health, Ministry of Public Health, Yaoundé, Cameroon; University of North Carolina at Chapel Hill, UNITED STATES

## Abstract

**Introduction:**

Meeting targets of the Sustainable Development Goals in the domain of maternal health and the Family Planning 2020 commitments for Cameroon requires an increased use of modern contraception. Long acting reversible contraceptives (LARCs) are methods which have been proven highly efficient with contraceptive failure rates of less than 1%. The **objective** of this survey was to determine the contraceptive method mix in the Biyem-Assi Health District and identify factors associated to the use of LARCs.

**Methodology:**

A cross-sectional community-based study was conducted from March 2015 to April 2015 targeting current female contraceptive users of childbearing age in the Biyem-Assi Health District. A multistep cluster sampling was used and data collected by trained surveyors using a pretested and validated questionnaire. Data were analysed using the statistical software Epi-Info version 3.5.4. Logistic regressions were used to identify associations between the use of LARCs and selected covariates and the strength of association measured with the odds ratio.

**Results:**

A total of 437 eligible women were included in the survey. Their mean age was 26.7±5.8 years and 45.8% were in a union. The contraceptive method mix decreased in this order; male condoms (76.0%), female condoms (7.6%), oral contraceptive pills (5.0%), implants (4.6%), and intrauterine devices (3.4%) giving us a LARC rate of 8%. Only 54.0% and 46.9% of the participants reported to be knowledgeable of the implant and intrauterine device respectively. Their contraceptive choices were determined principally by perceived efficiency and accessibility. The major factor significantly associated to LARC use was the number of living children above 2 (AOR = 3.90[1.53–9.94], p-value = 0.004). Though not statistically significant, associations were found between LARC use and other factors like marital status, level of education, religion and future fertility desire.

**Conclusion:**

The rate of use of LARCs is still very low among these women. The number of living children is significantly associated with the use of LARCs. The local family planning policy makers should intensify sensitization on the benefits and side effects of modern contraception and LARCs in order to create more awareness and improve contraceptive uptake.

## Introduction

It is no doubt today that a sustainable use of modern contraception is paramount in meeting the Sustainable Development Goal (SDG) targets set on maternal and neonatal morbi-mortality [1, 2]. Even though the rate of use of modern contraception in Africa [3] and more precisely in Cameroon [4] has experienced a slight increase over the last two decades (from 4%-14% between 1991 and 2011, and an increase from 12%-23% in Yaoundé between 1991 and 2011), the rate of unplanned pregnancy, induced abortions and maternal morbi-mortality remain relatively very high [4, 5, 6].

A good proportion of couples willing to limit or space births in the developing world, Africa [4] and more precisely in Cameroon still do not use contraception and even in settings where the unmet need for family planning has been brought down to low levels (or rate of contraceptive use high) [7], indicators of poor maternal health remain unacceptably high [4, 8]. Even if the rate of use of contraception and the unmet need for family planning are not the only indicators of poor maternal health, increasing modern contraceptive use and adherence can significantly reduce maternal morbi-mortality. According to the 2011 demographic and health survey, 26% of women and 15% of men in households have never been to school and only 38.6% in urban areas have acquired secondary education. The average number of people per household varies from 4.6 in rural areas to 5.4 in urban areas including both the nuclear and extended families. More than one quarter of households have children of below 18 years who do not live with their parents. Cameroon is made up of more than 230 ethnic groups. Yaoundé is the political capital of Cameroon and has a cosmopolitan population with more than 75% of the christian faith [4].

Cameroon with the aim of scaling up modern contraceptive use and meeting SDG targets agreed to number of commitments of the “Family Planning 2020” (FP2020) since 2014. FP2020 is a global partnership that supports the rights of women and girls to decide, freely, and for themselves, whether, when, and how many children they want to have [1]. As concerns commitments of the FP2020, the Government of Cameroon committed to: ensure contraceptive security; to avoid stock outs; provide the full range of contraceptive commodities by ensuring quality services; to improve on family planning counselling, training, and supervision of caregivers; and to ensure the government’s and its partner’s accountability for funding family planning programmes [1].

In this light, the Cameroon government took a financial commitment to conduct advocacy to step up the State‘s budgetary allocation for reproductive health to 5 percent yearly by 2020 and for family planning to 5 percent yearly by 2020. In addition, Cameroon committed to mobilize donors, including the private sector and civil society to finance family planning. Cameroon policy commitments of the FP2020 included but not limited to: ensuring the mobilization of the budget line for the actual purchase of contraceptive commodities; establish a mechanism to subsidize family planning services for the most vulnerable users; and to strengthen the multi-sectorial commitment to family planning.

It is clear that the correct use of modern contraception alone can help reduce maternal mortality by a third while improving on the family’s social and economic welfare [8, 9].The uptake of highly effective contraceptive methods which have been associated with high contraceptive continuation rates could significantly improve on the above two indicators. Long acting reversible contraceptives (LARCs) used in Cameroon include the intrauterine device and the hormonal implant. These methods of contraception are the most efficient reversible contraceptive services offered to clients with failure rates greatly less than 1%. Benefits associated with the use of these methods include; virtually no possibility of user’s error can prevent pregnancy for long periods of time once in place, and does not need to be put in place or needs any modification during the sexual act [2, 8, 10].

Long acting reversible contraceptive methods in Cameroon are offered to clients only in clinics and hospitals, integrated with other activities of the reproductive health package. The number of trained care givers in this field is still limited. However, the advent of many non-governmental family planning bodies and the efforts of the Cameroon health system has greatly reduced the cost of such highly efficient methods (LARCs however still remain relatively more expensive than the other reversible methods).

Despite the above advantages associated to LARCs, the rate of use of these very efficient contraceptive methods remains very low in many parts of Africa including Cameroon (In 2011, only about 1% and 0.2% of women in Yaoundé were using the implant and IUD respectively). [4]. The use of contraception in most developing countries is shifted towards the comparatively less efficient short acting barrier (condoms) and hormonal methods (like oral pills and injectable) [4, 8, 9, 11, 12, 13]. In Yaoundé, About 15.3%, 3.2% and 2.4% resorted to condoms, injectable and pills respectively in 2011 [4]. Many real and perceived surmountable obstacles could explain the low rate of use of LARCs. For instance, women have little awareness of the benefits of LARCs, women still carry lots of misperceptions about the risks of infection, infertility, and potential side effects such as irregular bleeding [14]. However modern intrauterine contraceptive methods have not been associated with infertility and are suitable for use in young women. In addition, continuity and satisfaction rates with all LARCs are high, indicating either a difficulty on demand in discontinuing these methods or the fact that their side effects might be acceptable for most users [10, 12, 13].

Most studies trying to address the use of LARCs have been carried out in Ethiopia; East of Africa with relatively similar sociodemographic characteristics. Literature on this subject matter in Cameroonis still sparse. The rate of unmet need for family planning among women in the Biyem-Assi Health District was 20.4% [7] and we sought to determine the contraceptive method mix with a focus on LARCs, identify contraceptive preferences and factors associated with the use of LARCs in this population. An understanding of these will help family planning policy makers plan interventions or redesign existing interventions to improve on the current contraceptive method mix in urban Cameroon.

## Methodology

### Ethics statement

The ethical approval for the study was obtained from the institutional ethical review board of the Faculty of Medicine and Biomedical Sciences of the University of Yaoundé I.

### Study design, setting, sample size, sampling and procedure

A cross-sectional community-based survey was conducted from March 2015 to April 2015 targeting current female contraceptive users (A woman who was using or had used a contraceptive method in the past one month before onset of the survey) of childbearing age in the Biyem-Assi Health District. One of the highest modern contraceptive prevalence during the 2011 National Demographic and Health Survey in Cameroon was recorded in Yaoundé [4] and our survey was carried out in one of its largest and most populated health districts; the Biyem-Assi Health District. The Biyem-Assi Health District is an urban health district made up of four health areas (Mendong, Melen, Mvog-Betsi, and Biyem-Assi) with a cosmopolitan population. We chose to conduct this study in urban settings where the availability of contraceptive methods in the market is not a problem. This was with an aim of limiting the influence of contraceptive inaccessibility on their contraceptive choices and preferences. Eligible participants were sexually active women of childbearing age living in the Biyem-Assi Health District who were current contraceptive users. Excluded were women who had adopted bilateral tubal ligation as their method of contraception. The minimum required sample size for the survey was estimated at 380 participants with the following parameters: the expected proportion of women adopting LARCs (13.1% reported in a similar Ethiopian population-based survey) [2], the absolute precision required on either sides of the proportion, threshold of error at 5% and a cluster effect of 2. A non-response rate of 10% adjusted our sample size to 418.

All four health areas were included in the survey and the total population of the health district partitioned into 70 geographical clusters and 50 randomly selected with replacement. Within a selected cluster, all road junctions were identified and one chosen randomly. By tossing a plastic bottle at a selected junction, a street was chosen and all households on the left hand side of the surveyor were systematically included in the survey. At the household level, all eligible and willing participants were included in the survey. The number of selected participants per geographical cluster was proportional to the estimated population of the cluster. Data at household level were collected by trained surveyors led by field supervisors using a pretested and validated questionnaire which they administered face to face. No incentives were given to participants during the survey. Their general characteristics, socio-demographic and economic variables **(**ge, marital status and number of years of cohabitation, religion, level of education, monthly revenue, and children alive), contraceptive use, preference, other determinants **(**last menstrual period, History of surgery, Coitarche, History of contraceptive use, Use of contraception and methods used, Method(s) used from first sexual contact, Reasons for the choice(s) were all collected in a questionnaire).

### Data analysis

After thorough verification and validation of filled questionnaires by the principal investigator (questionnaires that lacked very vital information like the age of participant, information on contraceptive use and LARCs precisely were excluded), the data were double entered into a pre-developed data capturing sheet, cleaned, compared, and analysed using the statistical software Epi-Info version 3.5.4. Analyses included amongst others; calculation of frequencies and their 95% confidence intervals for categorical variables, and means for continuous variables. The strength of association between the use of LARCs (women using either an intrauterine device or an implant) and the selected covariates (level of education, marital status, religion, number of living children, and future desire to give birth) was estimated by calculating odds ratios and their 95% confidence intervals. These covariates were selected based on previous literature in the subject matter. During multivariate analysis, a multiple logistic regression model was used to check for potential confounders which included level of education of the partner, age, and estimated monthly revenue. Each covariant was first of all introduced into the model alone to observe singular effect and later introduced in a multivariate model where all the other factors were simultaneously controlled to calculate the adjusted effect size (OR). All with a statistically significant threshold set at p-value ≤0.05.

### Ethical consideration

Non-respect of the participant’s autonomy and confidentiality were two potential ethical issues to consider during the survey. Questionnaire coding, and clear explanation of the consent sheet limited these two problems. In addition, only consenting women were included in the survey. For participants aged below 18 years, consent was obtained from their parents or their legal representatives. An ethical clearance was obtained from the institutional ethical review board of the Faculty of Medicine and Biomedical Sciences of the University of Yaoundé I. In addition, an administrative authorization was obtained from the Biyem-Assi District Health Service.

## Results and discussion

Our survey registered a non-response rate of 6.6%. A total of 437 participants were included in the survey with 2.7% of the sample aged below 18 years and therefore required consent from their legal representatives. The mean age of the 437 participants was 26.7±5.8 years and about 4 in every 10 women interviewed were in a union. The population was dominated by Christians (97.3%), and a few Muslims. About 8 in every 10 women had acquired at least a secondary education. Concerning their estimated monthly revenue, 63.2% earned less than fifty thousand FCFA (80.75 US dollars) in a month.

Table 1 below shows the use of each contraceptive method (contraceptive method mix) and their contraceptive preferences. The rate of use of the different modern contraceptive methods decreased in this order; male condoms (76.0%), female condoms (7.6%), oral contraceptive pills (5.0%), implants (4.6%), and intrauterine devices (3.4%). This order is similar to that reported in the Cameroon national demographic and health survey in which the male condom and intrauterine device were reported in same positions [4]. The contraceptive method mix of a population varies considerably from time to time based on a number of factors (the cultural beliefs on specific methods, the availability and accessibility of the different methods, the competence of the family planning team of the population….). In FP2020 countries, the modern contraceptive mix has been described to show significant variations. Even though there is no “ideal” method mix, it is generally accepted that improving access to a large variety of contraceptive methods will directly improve quality of care and ensure a full family planning choice from a rights-based framework. Increasing contraceptive variety within a population could lead to improved access and use of long acting and more effective contraception [13]. Among contraceptive users, the percentage practicing long acting reversible contraception was 8%. This is very low compared to a rate of 13.1% and 17.6% which were reported in 2014 and 2015 in two population-based surveys in Ethiopia respectively [2, 9]. This could be explained by the lack of sufficient awareness, on the pros and cons of these methods and lack of technical know-how among the family planning providers on these methods in our setting. Also, family planning services in most hospitals in our setting are not very effective and these methods are not offered out of family planning clinics.

**Table 1:** Contraceptive methods used and preferred.

	Contraceptive method Currently in use n(%)*	Most used contraceptive method from first sexual contact n(%)	Most Preferred contraceptive method: n(%)**
Male condoms	332(76.0)	338(77.3)	261(66.8)
Female condoms	33(7.6)	12(2.7)	9(2.3)
Implants	20(4.6)	13(3.0)	34(8.7)
Oral pills	22(5.0)	18(4.1)	18(4.6)
Intrauterine device	15(3.4)	12(2.7)	11(2.8)
Injectable	9(2.1)	9(2.1)	22(5.6)
Spermicides	3(0.7)	2(0.5)	2(0.5)
Diaphragms	1(0.2)	1(0.2)	1(0.3)
Cervical caps	1(0.2)	1(0.2)	0(0.0)
Coitus interruptus	21(4.8)	9(2.1)	6(1.5)
Fertility period based methods	44(10.1)	21(4.8)	24(6.1)
Lactation amenorrhoea method	4(0.9)	1(0.2)	1(0.3)
Others	2(0.5)	0(0.0)	2(0.5)
Total	437	437	391

*Note that in this case, somebody could be using more than one contraceptive method; only the most effective of these methods was reported.

**Here, not all the current contraceptive users could make a choice of their most preferred method.

Moreover, the knowledge of these women on contraception and more precisely on LARCs is still scanty; this was clear from the survey results which indicated that only 236 (54.0%) and 205 (46.9%) were familiar with the implant and intrauterine device respectively (not on the tables). This knowledge on LARCs is however relatively higher than that recorded in the 2011 demographic and health survey in which only 38.9% and 47.0% were familiar with the intrauterine device and implant respectively [4]. In addition, lots of perceived misperceptions have been associated with the intrauterine device (associated to cancer of the uterus and the cervix, and infertility) and the implants (can disappear into the body when placed under the skin, infertility) [10, 14]. The impression of a foreign object in the body could also contribute significantly towards reducing the demand for these contraceptive methods [14].

The rate of use of LARCs in this survey even though relatively low, is better compared to reports of the 2011 Cameroon National Demographic and Health Survey during which only 0.2% and 1.0% resorted to intrauterine devices and implants respectively in Yaoundé [4]. This could be explained by sample variation, the improvement in the delivery of family planning services over the years and the reduction in the cost of these methods of contraception through different government subsidies. Cameroon, a FP2020 commitment maker since 2014 remains one of the developing countries with a dying need of stepping up modern contraceptive use [1]. Looking at the results of this survey, the FP2020 commitments of the Cameroon government and the SDGs [1], it is clear that if Cameroon would meet the recommended targets on family planning and maternal morbi-mortality, strategies of counselling and focused population education on family planning methods need revisiting. More is still to be done in the domain of changing the false perceptions of these women vis-à-vis modern contraception and particularly these highly efficient forms. More general educational sessions needs to be organised and the women and their husbands counselled on the benefits of these methods which greatly surpass the surmountable side effects. The contraceptive mix was shifted towards the short acting barrier method in this population; the condom (83.6%). The relatively high rate of use of condoms is however not surprising given its dual property (prevention of unwanted pregnancy and sexually transmitted infections). Also, the media and public campaigns aimed at reducing the incidence of the human immunodeficiency virus have greatly increased the rate of condom use within the population. Even though the use of condoms constitutes one of the major strategies for prevention of sexually transmitted infections and the human immunodeficiency virus, the efficacy of this method is very low compared to the LARCs as far as contraception is concerned. The use of condoms for contraceptive purposes is generally associated with intermittent discontinuity either because of unavailability at the time of sexual contact or because of other partner dependent reasons. This increases its failure rates and exposes clients to undesired pregnancies, induced abortion and would go a long way in increasing maternal morbi-mortality.

A good proportion (15.8%) of users still relied on traditional contraception (counting of the cycle, lactation amenorrhoea method, and coitus interruptus) which is least effective given the many factors that can influence its efficacy.

These women however preferred these methods in the following order; male condoms (66.8%), implants (8.7%), injectable (5.6%), oral contraceptive pills (4.6%) and intrauterine devices (2.8%). From the results, it is clear that the position occupied by the intrauterine device both in use and preference is still a problem in this population. As already mentioned above, the real and perceived side effects which are well surmountable can explain this.

Table 2 shows some reasons for the above current contraceptive choices. Perceived efficacy (55.8%) and easy accessibility (28.4) were by far the two major reasons explaining their choices. According to analysis of the 1998 DHS data, method efficiency and accessibility were reported as major reasons determining contraceptive choices among women in Cameroon [15]. From these results, perceived efficiency is outstanding and is significantly affected by the quality of counselling which is given to the patients. Even though the efficacy of LARCs is widely accepted to be higher compared to that of other methods, the knowledge of the population on the other contraceptive methods and the fears of the side effects of LARCs seriously affect the contraceptive method mix. This indicates that much is left to be done in this domain to change the perceptions of these clients by giving them full and correct information about the different contraceptive methods.

**Table 2:** Reasons explaining the current contraceptive choices**.

Reasons	n (%)
It is effective	244(55.8)
It is easily accessibl	124(28.4)
It is cheap	85(19.5)
It is my partner’s choice	48(11.0)
It prevents sexually transmitted infections	43(9.8)
Prescribed by a medical personnel	226(5.9)
Others	63(14.4)

**It should be noted that participants could select more than one reason.

Table 3 shows the different factors associated to long acting reversible contraceptive practice. During univariate analysis, it was found that marital status (OR = 2.13[1.04–4.35], p-value = 0.038), and number of living children greater than 2 (OR = 2.87[1.25–6.52], p-value = 0.012) had significant statistical associations to the use of a long acting reversible contraceptive method. But when controlled for potential confounders in a multiple logistic regression model, it was found that only the number of living children greater than 2 (AOR = 3.90[1.53–9.94], p-value = 0.004) had an independent and statistically significant association with long acting reversible contraceptive practice.

**Table 3:** Determinants of long acting reversible contraceptive practice.

Factors	Univariate analysis			Multivariate analysis		
	OR	CI 95%	p-value	Adj OR	Adj CI 95%	p-value
Women in union(Y/N)	2.13	1.04–4.35	0.037*	1.90	0.89–4.05	0,099
Level of education above primary (Y/N)	0.61	0.25–1.46	0.265	0.70	0.28–1.77	0.452
Future desire to give birth(Y/N)	0.46	0.20–1.07	0.071	0.59	0.21–1.62	0.304
Religion as catholic (Y/N)	1.56	0.77–3.16	0.277	1.39	0.66–2.95	0.39
Number of children alive greater than 2 (Y/N)	2.87	1.25–6.52	0.012*	3.90	1.53–9.94	0.004*

Y/N = Yes/No, OR = Odds Ratio, Adj OR = Adjusted Odds Ratio, CI = Confidence Interval, Adj CI: Adjusted Confidence interval,

* Statistically significant (p-value ≤ 0.05)

This implies that a woman with more than 2 living children was three to four times more likely to adopt a LARC than a woman with fewer children. This finding was in order with that reported in two Ethiopian surveys which indicated that as the number of living children increased, the women were significantly more likely to adopt the long acting reversible contraception [8, 11, 14]. Because of the perceived association of infertility with these methods, women who do not yet have children or have not completed childbearing turn to avoid these methods of contraception [14]. Unplanned pregnancy however knows no age, does not take into account the number of previous pregnancies and the number of living children. Counselling on the adoption of LARCs should therefore put more emphases on women with fewer children while putting forth clearly the benefits and side effects of the different methods to allow for the selection of an adequate method for the woman.

### Limits and strength of the survey

The results herein should be interpreted with care. Our study does not extend to give a picture on the long acting irreversible surgical methods and hormonal emergency contraceptive use. Potential contra-indications to LARCs were not evaluated, as this could partly explain the low rate of use. In addition, our study was carried out only in an urban setting; contraceptive behaviours in the rural and urban settings may show significant discrepancies. Contraceptive availability was not evaluated in the different family planning units; past counselling sessions were not evaluated and participants were not scored using the method information index. Contraceptive continuation rates cannot be evaluated with our survey as a standard. Moreover, the cross sectional design of this survey only permits emission of hypotheses between variables but no cause-effect relationships can really be established. Some relationships established between variables might just be temporary. However, the study is designed with adequate and adapted methodology to check for all forms of bias and can serve to give a pictorial view of the situation on the contraceptive method mix with a focus on LARCs in the Biyem-Assi Health District.

## Conclusion

The use of contraceptive methods in the Biyem-Assi Health District-Yaoundé is still highly tilted towards the short acting barrier methods. The rate of use of LARCs among these women is still very low. The contraceptive choices of this population are determined principally by their perceived efficacy and accessibility. The major determining factor significantly associated to LARC use is the number of living children above 2. Though not statistically significant, associations were found between LARC use and other factors like marital status, level of education, religion and future fertility desire. More focused sensitization is required with more emphasis put on LARCs to increase the rate of use in this population. More compelling and large scale studies should be designed to better understand the drivers of the particular method mix in Cameroon and dig deeper into the reasons why LARCs are underutilized.

## Supporting information

S1 File Data base evaluating contraceptive needs. (MDB)

## Abbreviations

LARCs: Long Acting Reversible Contraceptives
IUD: Intrauterine Devise
SDG: Sustainable Development Goals
FP2020: Family Planning 2020

## References

[1] Dockalova B., Lau K., Barclay H., Marshall A. Sustainable Development Goals and Family Planning 2020. The International Planned Parenthood Federation (IPPF). United Kingdom; June 2016: p 1–12 https://www.ippf.org/sites/default/files/2016-11/SDG%20and%20FP2020.pdf. Accessed 04 June 2018.

[2] Getinet S, Abdrahman M, Kemaw N, Kansa T, Getachew Z, Hailu D, et al Long Acting Contraceptive Method Utilization and Associated Factors among Reproductive Age Women in Arba, Minch Town, Ethiopia. Greener J Epidemiol Public Heal. 2014;2(1):23–31.

[3] Gebeyehu WA, Assefa TG, Zeleke AA. Trends and Determinants of Contraceptive Use among Young Married Women (Age 15–24) Based on the 2000, 2005, and 2011 Ethiopian Demographic and Health Surveys: A Multivariate Decomposition Analysis. Rockville, Maryland, USA; Report No.: 103.

[4] Institut National de la Statistique(INS) et ICF. Planification familiale. In: Enquête Démographique et de Santé et à Indicateurs Multiples 2011. Calverton, Maryland, U.S.A; 2011. p. 99–117.

[5] National Institute of Statistics/UNDP. COUNTRY REPORT ON PROGRESS IN ACHIEVING THE MILLENIUM DEVELOPMENT GOALS. 2008. Cameroon. http://planipolis.iiep.unesco.org/upload/Cameroon/CameroonMDG2008.pdf. Accessed Dec 2015

[6] Warriner IK, Shah HI. Preventing Unsafe Abortion and its Consequences; Priorities for Research and Action New York: Guttmacher Institute; 2006.

[7] Ajong AB, Njotang PN, Yakum MN, Essi MJ, Essiben F, Eko FEE, et al Determinants of unmet need for family planning among women in Urban Cameroon: a cross sectional survey in the Biyem-Assi Health District, Yaoundé. BMC Women’s Health. 2016;16(4):1–8. 10.1186/s12905-016-0283-9. 26791410 PMC4721192

[8] Teferra AS, Wondifraw AA. Determinants of long acting contraceptive use among reproductive age women in Ethiopia: Evidence from EDHS 2011. Sci J Public Heal. 2015;3(1):143–9.

[9] Sahilemichael A, Temesgen K, Gemechukejela. Determinants of Long Acting Reversible Contraceptives Use among Child Bearing Age Women in Dendi District, Western Ethiopia. Women ‘ s Heal Care. 2015;4(4):1–4.

[10] Gudaynhe SW, Zegeye DT, Asmamaw T, Kibret GD. Factors Affecting the use of Long-Acting Reversible Contraceptive Methods among Married Women in Debre Markos Town, Northwest Ethiopia 2013. Glob J Med Res E Gynecol Obstet. 2014;14(5):8–15.

[11] Earsido A, Gebeyehu A, Kisi T. Determinants of Long Acting and Permanent Contraceptive Methods Utilization among Married Women in Hossana Town, Southern Ethiopia: J Preg Child Heal. 2015;2(3):1–6.

[12] Bulto GA, Zewdie TA, Beyen TK. Demand for long acting and permanent contraceptive methods and associated factors among married women of reproductive age group in Debre Markos Town, North West Ethiopia. BMC Womens Health. 2014;14(1):1–12. 24625360 10.1186/1472-6874-14-46 PMC3975156

[13] Seiber EE., Bertrand JT., Sullivan TM., Changes in Contraceptive Method Mix In Developing Countries. International Family Planning Perspectives. 2007; 33(3):117–123 10.1363/ifpp.33.117.07 17938094

[14] Tebeje B, Workneh D. Prevalence, Perceptions and Factors Contributing to Long Acting Reversible Contraception Use among Family Planning Clients, Jimma Town, Oromiya Region, South-West Ethiopia. J Women's Health Care. 2017; 6(1): 1–10. 10.4172/2167-0420.1000351

[15] Akam E. Les facteurs de la contraception au Cameroun au tournant du siècle. Analyse des données de l’enquête démographique et de santé de 1998. Paris: GrippsLa planification familiale en Afrique. Documents d’analyse n° 6; 2005. 1–62 p. http://www.ceped.org/cdrom/gripps/pdf/cameroun.pdf. Accessed 06 June 2018.

